# A Novel Diabetic Murine Model of *Candida albicans*-Induced Mucosal Inflammation and Proliferation

**DOI:** 10.1155/2014/509325

**Published:** 2014-02-18

**Authors:** Tomoya Sano, Kiyokazu Ozaki, Yui Terayama, Yasushi Kodama, Tetsuro Matsuura

**Affiliations:** ^1^Department of Pathology, Faculty of Pharmaceutical Sciences, Setsunan University, Hirakata, Osaka 573-0101, Japan; ^2^Laboratory of Clinicopathological Therapeutics, Faculty of Pharmaceutical Sciences, Hiroshima International University, Kure, Hiroshima 737-0112, Japan

## Abstract

Chronic hyperplastic candidiasis (CHC) lesions will progress to dysplasia with some of these developing squamous cell carcinoma (SCC). It is well known that diabetic patients are predisposed to candidiasis. Previously, we found that alloxan-induced diabetic rats spontaneously have mucosal hyperplasia with *C. albicans* infection and that those lesions progress to SCC. Here, we developed a rat model of candidiasis with diabetes progressing to mucosal proliferation. Diabetes was induced in thirty rats by single intravenous administration of alloxan. Ten nondiabetic rats and fifteen diabetic rats then received *C. albicans* containing solution orally, and additional fifteen diabetic rats received saline in the same manner. The administration of *C. albicans* induced mucosal candidiasis and the related mucosal hyperplastic changes in all the diabetic rats and progressed to SCC in one rat. Chronic suppurative inflammation of the mucosa developed in the forestomach with infection by *C. albicans*. The same lesions were only detected in the forestomach of 4 diabetic rats without *C. albicans* treatment. After *C. albicans* treatment, none of the nondiabetic rats showed mucosal changes or fungus infection in the forestomach. These findings demonstrate that a prolonged diabetic condition can cause *C. albicans* infection and enhance *C. albicans*-related mucosal hyperplasia.

## 1. Introduction

*Candida* species, especially *Candida albicans *(*C. albicans*), are one of the most common human pathogens with the possibility of causing a variety of mucosal lesions in the upper alimentary organs and vagina. *Candida* infections are associated with the administration of antibiotics, steroids, immunosuppressive agents, and myeloablative radiation therapy [1]. Other risk factors for* Candida* infection are diabetes, acquired immunodeficiency syndrome (AIDS), and iron and vitamin deficiency. *Candida* infection of the human oral mucosa not only causes chronic hyperplastic candidiasis, characterized by thickening of the epithelium associated with acute and chronic inflammation, but might also lead to malignant change [1–3]. A number of clinical relevant rodent models of mucosal candidiasis have been established to study host-pathogen interactions and antifungal drug and/or probiotic efficacy. It is well known that the oral and/or gastrointestinal candidiasis is induced by experimental administration of *C. albicans* in rats [4–6]. However, mucosal infection models generally require the use of immunosuppressive agents or antibiotics [4–6]. In addition, these rodent models of candidiasis are not capable of inducing severe mucosal proliferative lesions.

Previously, we reported that alloxan-induced diabetic rats frequently have severe mucosal proliferative lesions with fungus and bacterial infections in the forestomach and that these lesions progress to squamous cell carcinoma (SCC) [7]. Antidiabetic and antifungal treatment reduced the degree of these changes [7–9]. On the other hands antibiotic treatment increased the incidence of proliferative lesions with *C. albicans* [10]. Thus, we revealed that proliferative changes were markedly associated with infection by *C. albicans*. However, it is difficult to know when these changes occurred in our previous study. In addition, the induction of severe proliferative changes took at least a year in our previous reports [7–9].

In this study, we evaluate the diabetic rodent model of candidiasis histologically and the role of the diabetic condition in the onset of *C. albicans* infection in alloxan-induced diabetic rats.

## 2. Materials and Methods

### 2.1. Animals and Diets

Female WBN/Kob rats were obtained from Japan SLC, Inc. (Shizuoka, Japan). They were reared in a barrier-sustained animal room maintained at a temperature of 24 ± 2°C and a relative humidity of 60 ± 20%, with 12 h light/dark cycles, and ventilated at least 12 times/h with sterilized fresh air. All the rats were housed and reared in aluminum mesh cages. To protect against infection, the cages were changed once or more each week. Rats were given a pelleted diet (CRF-1; Oriental Yeast, Tokyo, Japan).

The study was approved by the Committee for Animal Experiments of Setsunan University.

### 2.2. Glycosuria and Glycemia Monitoring

Fresh urine samples were collected in metabolism cages. Urinary glucose levels were measured semiquantitatively, using a urine test paper (Wako Pure Chemical Industries, Osaka, Japan) every day from day 1 to day 3 after alloxan dosing, once every week for 1 month after the first week, and once every month thereafter from the fresh urine samples obtained from alloxan-induced diabetic rats. Blood glucose levels were also measured semiquantitatively by the glucose oxidase method (Glutest E; Sanwa Kagaku, Aichi, Japan) once every month from the fourth week after dosing, using blood samples from the tail vein. Samples of blood from the tail vein and fresh urine were collected from 1:00 to 4:00 pm.

### 2.3. Experimental Design

A total of 40 female WBN/Kob rats were divided into three groups at 10 weeks of age. Thirty rats, aged 10 weeks, were given a single dose of alloxan (Sigma-Aldrich Japan, Tokyo, Japan) via the tail vein at a dosage level of 40 mg/kg body weight. The concentrations were set up as a given dose according to which a rat can survive for a long period of time after developing signs of diabetes and which induces continuous glycosuria.

A strain of *C. albicans,* which was obtained from a rat forestomach with proliferative change in our previous study, was used for the inoculations. A slope of potato dextrose agar was streaked with organisms 72 hr before inoculation and allowed to incubate at room temperature (23°C). The yeast cells were rinsed from the slope with saline and suspended at a concentration of approximately 5 × 10^6^ CFU/mL. A 1 mL volume of this suspension was used for oral treatment on three alternate days during the first 2 weeks of the study and thereafter once in a week. Ten nondiabetic female WBN/Kob rats (C group) and 15 alloxan-induced diabetic rats were given this suspension (AC group) for 10 weeks from 12 weeks of age. The remaining 15 alloxan-induced diabetic rats (AL group) received saline in the same manner.

All rats of the AL, AC, and C groups were given chlorinated water and fed diet *ad libitum*. In this study, the rats of the AC and C groups were reared separately from the rats of the AL group. The rats were sacrificed at the 37th week of age.

### 2.4. Histopathological Analysis

One moribund animal (a rat of the AL group) was sacrificed at 14 weeks of age. The remaining 39 rats were killed by exsanguination from the abdominal aorta under deep anesthesia at the end of each scheduled period. The entire alimentary tract was immediately removed following necropsy. The organs of the 39 rats were immersed in 10% phosphate-buffered formalin solution immediately after necropsy.

The fixed organs were trimmed, dehydrated by an automated processor, and embedded in paraffin wax. Sections (4 *μ*m thick) of tissue specimens were stained with hematoxylin-eosin for histopathological examination.

The severity of the proliferative lesions in the forestomach squamous epithelium was judged from the thickness of the mucosal epithelium described in the previous report as follows: −, equivalent with control; +, mild change; ++, moderate change; +++, severe change [7–9]. The suppurative inflammation was graded as +, focal change; ++, multifocal changes; ++, diffuse change. The chronic inflammation was graded as +, focal change; ++, diffuse change.

### 2.5. Immunohistochemical Analysis

Immunohistochemical confirmation of *C. albicans* and cell proliferation was conducted on representative forestomach sections. The sections were deparaffinized in xylene and rehydrated through graded ethanol at room temperature. Rehydrated sections were digested by pepsin for 20 min at 37°C to retrieve the antigen. Solutions and washes were prepared between the various steps using 0.05 M Tris-buffered saline (TBS, pH 7.6) with 0.01% Tween 20. Nonspecific endogenous peroxidase activity was blocked by exposure to 0.03% hydrogen peroxide in 100% methanol for 5 min, and masking was conducted with 1% bovine serum albumin in phosphate-buffered saline for 5 min at room temperature. Incubation was carried out overnight at 4°C with anti-*C. albicans* (diluted 1 : 400, MAB806; Chemicon, USA) mouse monoclonal antibody or anti-Ki-67 rabbit monoclonal antibody (diluted 1 : 500, SP6; Epitomics, USA). The slides were subsequently rinsed with TBS plus Tween 20, treated for 30 min at room temperature with Histofine Simple Stain rat MAX PO (M) (Nichirei, Tokyo, Japan) or a staining system kit DAKO LSAB 2 kit/HRP (DAKO Japan, Kyoto, Japan), rinsed with TBS plus Tween 20, incubated in diaminobenzidine solution containing 0.01% hydrogen peroxide for the peroxidase coloring reaction, and counterstained with Mayer's hematoxylin. Staining was negatively controlled by substituting mouse and rabbit isotype immunoglobulin, diluted to the same concentration, for the primary antibody.

### 2.6. Statistical Analysis

Fisher's exact test and the unpaired Student's *t*-test were used for statistical analysis of the body weight data. The Mann-Whitney *U* test was used to compare the histopathological findings. When the calculated *P* value was less than 0.05, the difference was considered statistically significant. Statistical analyses were performed using the StatMate III program (ATMS, Tokyo, Japan).

## 3. Results

### 3.1. Physical Condition of the Rats and Monitoring of Glycosuria and Glycemia

Severe hyperglycemia (>300 mg/dL) and glycosuria (>500 mg/dL) continued for 27 weeks from the day of alloxan injection to the time of scheduled necropsy in almost all the alloxan-treated rats (AL: 13/14, AC: 13/15). Two rats of the AC group and one rat of the AL group showed normal to mild hyperglycemia and glycosuria. The body weights of all the rats in the AL and AC groups decreased within several days of injection and the average body weight of these two groups was decreased compared to the C group (C: 237.7 ± 16.2 g, AL: 182.2 ± 15.4 g, and AC: 198.7 ± 19.8 g resp.).

### 3.2. Histopathology

**Figure 1:**
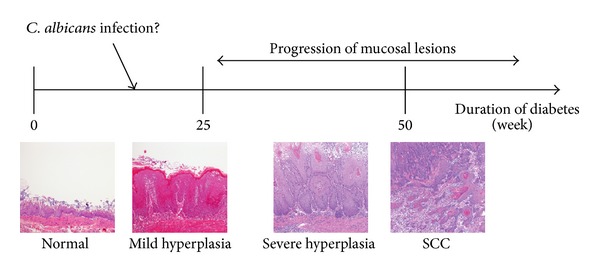
Schematic depicting the approximate time course of forestomach lesion development.

**Figure 2:**
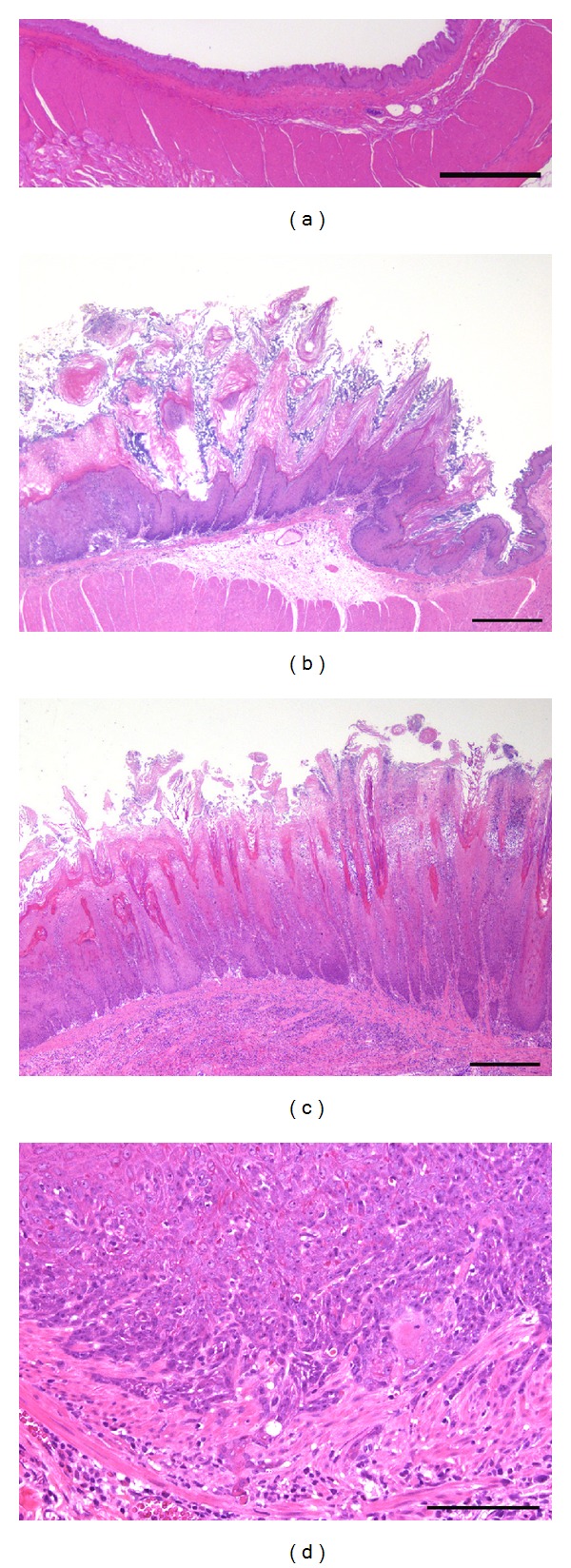
Mucosal hyperplasia and squamous cell carcinoma induced by *C. albicans.* (a) Normal forestomach mucosa in the C group. Scale bar, 500 *μ*m. (b) Mild hyperplasia of mucosal squamous epithelium in the AL group. Scale bar, 500 *μ*m. (c) Severe hyperplasia of forestomach mucosal squamous epithelium in the AC group. Neutrophils accumulate in surface mucosal epithelial layer, and lymphocytes and plasma cells infiltrate throughout the entire submucosal layer. Scale bar, 500 *μ*m. (d) Squamous cell carcinoma of the forestomach in the AC group. Scale bar, 100 *μ*m.

**Figure 3:**
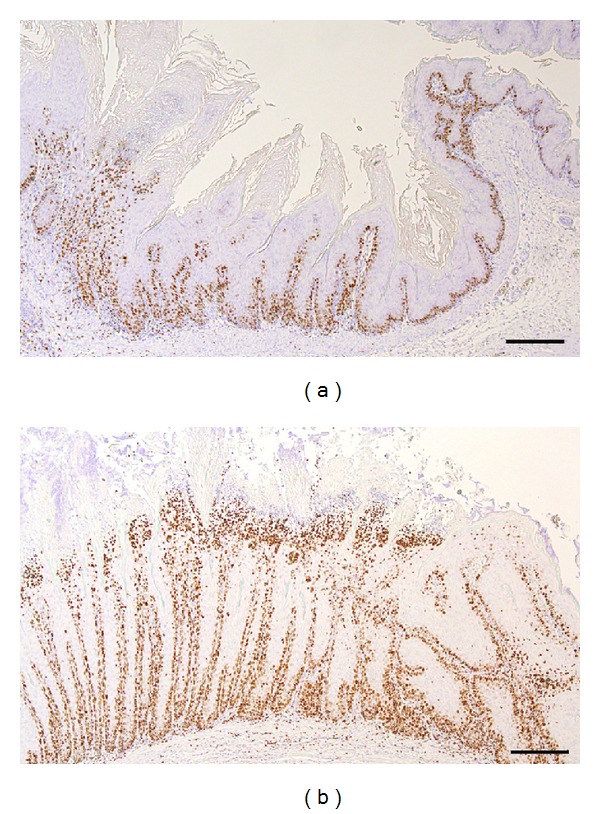
Cellular proliferation of mucosal epithelium induced by *C. albicans.* The Ki-67 positive cells of mucosal epithelium are increased in the AL group (a) compared to the AC group (b). Scale bar, 200 *μ*m.

**Figure 4:**
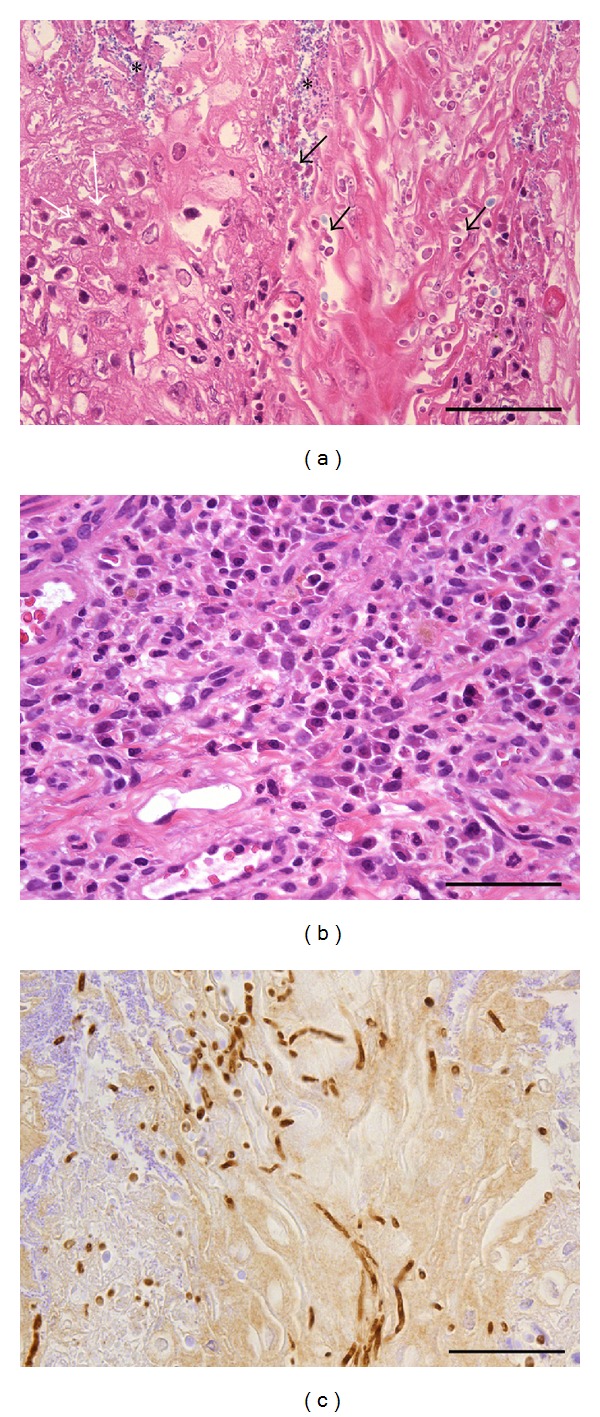
Mucosal and submucosal inflammation induced by *C. albicans.* (a) Neutrophils infiltrate (white arrows) in the hyperplastic mucosal surface with fungal (black arrows) and bacterial infection (asterisk) in the AC group. (b) Lymphoplasmacytic cell infiltration in the submucosa are observed in the AC group. (c) Fungi positive for a *C*.* albicans* antigen by immunohistochemical staining are seen in hyperplastic mucosal surface. Scale bar, 50 *μ*m.

Squamous hyperplastic changes in the forestomach were detected in the AL and AC groups, but not in the C group (Figure 2) (Table 1). The incidence and severity of the proliferative lesions were significantly enhanced in the AC group compared to the AL group (Table 1). Mild to moderate proliferative changes were only detected in the forestomach of 4 rats (28.6%) in the AL group, whereas all the rats in the AC group developed mild to severe squamous cell hyperplasia and this progressed to SCC in one case in the AC group (Figure 2) (Table 1). The absolute number of Ki-67 positive cells of the forestomach was also increased in the AC group compared to the AL and C groups (Figure 3). Two types of inflammatory change were observed in the hyperplastic mucosa (Figures 1(b) and 1(c)). Almost all of *C. albicans* infected animals from AL and AC groups have an accumulation of neutrophils in the surface of mucosa and lymphoplasmacytic cell infiltration in the submucosa (Figure 4). The incidence and severity of the inflammatory lesions were significantly enhanced in the AC group compared to the AL and C group (Table 2). Squamous cell hyperplasia and inflammatory changes were diffuse and were the most prominent changes observed on the side of the limiting ridge of the stomach in the AL and AC groups. However, no inflammatory changes were observed in the C group (Table 2). Ulcerated mucosal epithelium with bacterial or fungal infection was observed in the AL and AC groups. Filamentous fungi showing dimorphism such as yeast forms and mycelial forms were also frequent in the superficial mucosal layer in almost all the diabetic rats of the AC group (13/15, 86.7%) and 4 rats of the AL group (4/14, 28.6%). All of the fungi in the hyperkeratotic lesions of the mucosa were positive for *C. albicans* antibody in the 4 rats of the AL and the 13 rats of the AC groups (Figure 3). In addition, rod-shaped Gram-positive bacteria were also seen on the mucosal surface in all rats with or without hyperplasia. After treatment with* C. albicans*, none of the rats in the C group showed inflammatory changes or fungus infection in the forestomach.

## 4. Discussion

We have reported in our previous report that alloxan-induced diabetic rats exhibit mucosal proliferative lesions with chronic inflammation, and some lesions progress to SCC in the forestomach after 50 weeks with the diabetic condition [7–9]. Diabetes and *C. albicans* infection are profoundly involved in the pathogenesis of these changes. The present study revealed that inoculation of *C. albicans* quickly and unexceptionally induced *C. albicans* infection and its related mucosal inflammatory and proliferative changes in all the alloxan-induced diabetic rats. On the other hand, neither inflammation nor *C. albicans* infection were observed in the nondiabetic rats. These results were similar to that with uncontrolled diabetes mellitus which is well known to predispose individuals to oral candidiasis [11]. In addition, SCC was observed in one case of the AC group. Russel and Jones reported the histological character of oral candidiasis in rats [12]. Even though their model had a much longer experimental duration than our study, the affected rats showed severe hyperplasia but not SCC [12]. Thus, forestomach mucosal epithelium may have greater sensitivity than oral mucosal epithelium.

In this study, the *C. albicans* infected mucosal surfaces had severe inflammatory changes which were characterized by extensive neutrophilic infiltration. Neutrophils are known to be able to directly kill *Candida* through ingestion and killing or through neutrophilic extracellular traps [13]. This inflammatory response must indicate a response against the *C. albicans* infection. Diabetic patients have decreased oxidative killing ability of their neutrophils, so diabetics may not be able to eradicate pathogens as well as nondiabetics [14–16]. Thus, a long lasting diabetic state allowed infections and contributed to the persistence of *C. albicans* infection in this study. In the nondiabetic control group, none of the rats showed a hyperplastic mucosa with *C. albicans* infection or inflammatory changes. These results indicate that diabetes induced the *C. albicans* infection and the mucosal proliferation in our model. Furthermore, it has been reported that diabetes mellitus is one of the risk factors for some tumors in humans (liver, pancreas, endometrium, large intestine, breast, and bladder) [17]. A possible mechanism of tumorigenesis is considered diabetes including hyperinsulinemia, hyperglycemia, and inflammation. However, the risk of prostate cancer is reduced by diabetes [17]. In our previous study, noninsulin dependent diabetes enhanced genotoxic compound-induced forestomach squamous cell carcinoma [18]. Thus, both *C. albicans* infection and diabetes are considered to play an important role in the forestomach mucosal lesions.

Recently, special attention has been focused on the inflammation-related promotion of gastrointestinal cancer such as colorectal cancer and gastric cancer, and it was proven that chronic inflammation causes malignant tumors [19, 20]. The proliferation of the mucosa was thought to be related to the inflammatory changes in our study. Thus, *C. albicans* infection may stimulate the normal epithelium to initiate inflammation and/or promote mucosal proliferation. However, an increased incidence of SCC was not observed in this study. In addition, the animals, which had severe mucosal proliferation and/or SCC, were also infected with several kinds of bacteria (*Lactobacillus* sp., *Bacillus licheniformis*, and *Escherichia coli*) in our previous study. Thus, these coinfections may play an important role in the proliferative change.

Ki-67 is a cell cycle associated nuclear protein, used as a proliferation marker and corelated with malignancies [21]. Some of the literature showed an increase in Ki-67 expression according to the severity of epithelial dysplasia in the upper gastrointestinal tract [22, 23]. In our study, the Ki-67 positive cells of the forestomach increased with the severity of the hyperplasia and were enhanced in the AC group. These data might support that *C. albicans* infection plays an important role in the mucosal proliferation.

It became clear that prolonged uncontrolled diabetes can easily and unexceptionally facilitate *C. albicans* infection and enhance *Candida*-related mucosal proliferation in alloxan-induced diabetic rats. Furthermore, this experimental *C. albicans* infection model is useful for identifying the mechanisms involved in host-*C. albicans* interactions. In the future, we have to demonstrate the involvement of bacterial infections in the onset of the SCC and evaluate the genetic and/or epigenetic alterations in our model.

## Figures and Tables

**Table 1:** Histopathologic findings of hyperplastic changes in the forestomach.

	AC	AL	C
Effective number of rats	15	14	10
Squamous cell carcinoma	1 (6.6%)	0	0
Squamous cell hyperplasia	14 (93.3%)^∗∗##^	4 (28.6%)^##^	0
+	2 (13.3%)	2 (14.3%)	
++	8 (53.3%)	2 (14.3%)	
+++	4 (26.7%)	0	

**Significantly different from AL group, *P* < 0.01.

^##^Significantly different from C group, *P* < 0.01.

**Table 2:** Histopathologic findings of inflammatory changes in the forestomach.

	AC	AL	C
Effective number of rats	15	14	10
Suppurative inflammation in mucosa	13 (86.7%)^∗∗##^	4 (28.6%)^##^	0
+	2 (13.3%)	1 (7.1%)	
++	6 (40%)	3 (21.4%)	
+++	5 (33.3%)		
Chronic inflammation in submucosa	13 (86.7%)^∗∗##^	4 (28.6%)^##^	0
+	9 (60%)	4 (28.6%)	
++	4 (26.7%)	0	
*C. albicans* infection	13 (86.7%)^∗∗##^	4 (28.6%)^##^	0
Bacterial infection	15 (100%)	14 (100%)	10 (100%)

**Significantly different from AL group, *P* < 0.01.

^##^Significantly different from C group, *P* < 0.01.
